# A Plasma 5-Marker Host Biosignature Identifies Tuberculosis in High and Low Endemic Countries

**DOI:** 10.3389/fimmu.2021.608846

**Published:** 2021-02-24

**Authors:** Bih H. Chendi, Candice I. Snyders, Kristian Tonby, Synne Jenum, Martin Kidd, Gerhard Walzl, Novel N. Chegou, Anne M. Dyrhol-Riise

**Affiliations:** ^1^Institute of Clinical Medicine, Faculty of Medicine, University of Oslo, Oslo, Norway; ^2^Division of Molecular Biology and Human Genetics, Department of Science and Technology-National Research Foundation (DST-NRF) Centre of Excellence for Biomedical Tuberculosis Research, Faculty of Medicine and Health Sciences, South African Medical Research Council Centre for Tuberculosis Research, Stellenbosch University, Cape Town, South Africa; ^3^Department of Infectious Diseases, Oslo University Hospital, Oslo, Norway; ^4^Department of Statistics and Actuarial Sciences, Centre for Statistical Consultation, Stellenbosch University, Cape Town, South Africa

**Keywords:** tuberculosis, biomarkers, diagnosis, treatment response, endemic settings, biosignatures

## Abstract

**Background:** Several host inflammatory markers have been proposed as biomarkers for diagnosis and treatment response in Tuberculosis (TB), but few studies compare their utility in different demographic, ethnic, and TB endemic settings.

**Methods:** Fifty-four host biomarkers were evaluated in plasma samples obtained from presumed TB cases recruited at the Oslo University Hospital in Norway, and a health center in Cape Town, South Africa. Based on clinical and laboratory assessments, participants were classified as having TB or other respiratory diseases (ORD). The concentrations of biomarkers were analyzed using the Luminex multiplex platform.

**Results:** Out of 185 study participants from both study sites, 107 (58%) had TB, and 78 (42%) ORD. Multiple host markers showed diagnostic potential in both the Norwegian and South African cohorts, with I-309 as the most accurate single marker irrespective of geographical setting. Although study site-specific biosignatures had high accuracy for TB, a site-independent 5-marker biosignature (G-CSF, C3b/iC3b, procalcitonin, IP-10, PDGF-BB) was identified diagnosing TB with a sensitivity of 72.7% (95% CI, 49.8–82.3) and specificity of 90.5% (95% CI, 69.6–98.8) irrespective of geographical site.

**Conclusion:** A 5-marker host plasma biosignature has diagnostic potential for TB disease irrespective of TB setting and should be further explored in larger cohorts.

## Introduction

An estimated 10 million people were reported to have tuberculosis (TB) and nearly 1.5 million died of the disease in 2018 (1). New tools for TB diagnosis and monitoring of treatment responses are needed, particularly in resource-constrained settings (2). The limitations of sputum smear microscopy and sputum culture are widely published (2–5). Culture conversion after 2 months of TB treatment is mostly used when monitoring treatment response in clinical trials but has limited utility in individual patients (5, 6). Also, smear microscopy and the Xpert MTB/RIF tests are not suitable for TB treatment monitoring purposes as they cannot discriminate between dead and live bacteria (6–8). Thus, there has been an intensified search for suitable host immune biomarkers for TB diagnostics and monitoring treatment response.

Several studies that made use of specimens collected in Africa or other high TB burden settings have identified promising biomarkers in serum or plasma (9–11), *M.tb* antigen-stimulated blood (12–14), and other bodily fluids including saliva and urine (5, 15–17). Other studies conducted in high income/low endemic settings aiming to differentiate active TB from latent TB infection (LTBI) irrespective of HIV status, and for evaluating TB treatment (18–21) led to the identification of interferon-gamma inducible protein (IP)-10 as a candidate biomarker for TB diagnosis. Still, despite the numerous promising biomarkers identified so far only interferon-gamma (IFN-γ) release assays (IGRA) currently exist in clinical practice, but IGRAs do not distinguish active TB from LTBI (22) and are not useful in high burden settings (23). As highlighted in a recent report, host biomarker-based studies are often poorly designed and promising biomarkers are mostly evaluated at single-sites, without independent validation cohorts (24). Therefore, new studies evaluating promising biomarkers in multiple independent cohorts including participants recruited in both low and high endemic settings are needed (4, 25–27).

In the current study, we evaluated the potential utility of previously published plasma-derived biomarkers for TB diagnosis and monitoring of treatment response in adults with suspected active TB from low endemic (Norway) and high endemic (South Africa) settings.

## Methodology

### Study Participants

**Figure 1:**
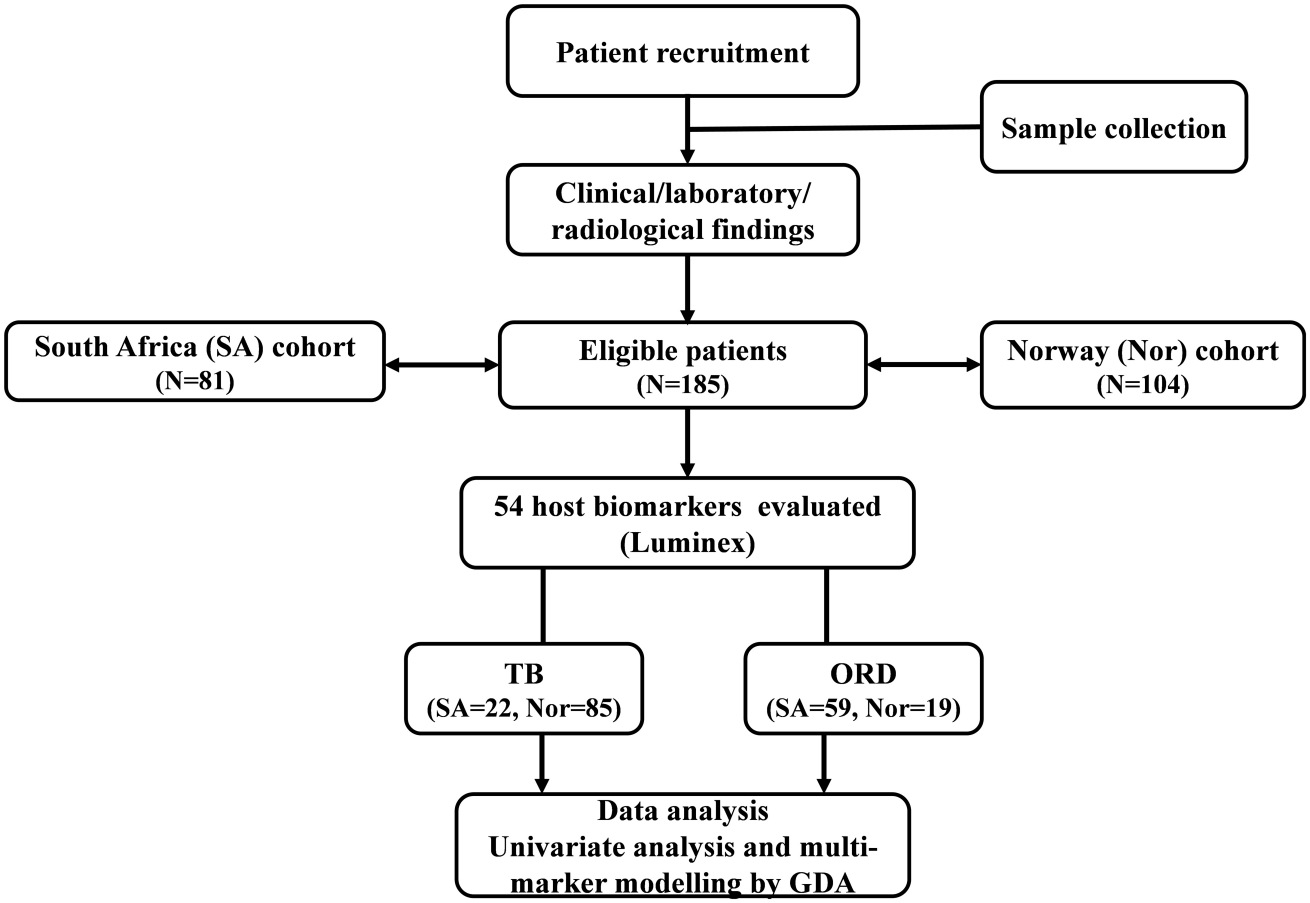
STARD diagram showing the study design and classification of study participants. TB, Tuberculosis cases; ORD, Individuals presenting with symptoms and investigated for pulmonary TB but in whom TB disease was ruled out; ROC, Receiver operator characteristics; GDA, General discriminant analysis.

Participants were recruited through longitudinal observational cohort studies at the Department of Infectious Diseases, Oslo University Hospital (OUH), Norway (*Prognostic Immunological markers in Tuberculosis)* from 2012 to 2019, and the Fisantekraal Clinic, a peripheral level health care center in the outskirts of Cape Town, South Africa; a field site for a larger biomarker study (*ScreenTB* project) from 2016 to 2019 (Figure 1).

Briefly, Norwegian study participants were patients admitted for medical evaluation on suspicion of having active TB. Adults with active TB and consenting to participate were recruited into the observational cohort. Medical history including co-morbidities and HIV co-infection were registered at inclusion. Clinical examination and chest X-rays were performed, and if indicated, supplemented with further radiological and/or histological investigations. TB diagnosis was based on either positive *Mtb* culture/ PCR or clinical diagnosis based on symptoms, radiological findings, and histology consistent with TB where anti-TB-therapy was started. Active TB patients were further categorized into pulmonary TB (PTB), extrapulmonary TB (EPTB) or combined (PTB + EPTB). TB patients were followed up with new visits at week 2, month 2, and month 6 after initiation of TB treatment. All patients were clinically cured at the end of treatment. Participants grouped as other respiratory diseases (ORD) were recruited from patients with symptoms of lower respiratory infections admitted to OUH in the same period.

South African study participants self-presented at the clinic with symptoms requiring investigation for active PTB and were recruited prior to the diagnosis of TB or ORD. TB was confirmed or ruled out using a combination of clinical, laboratory, and radiological findings as described in previous reports (9, 10). All individuals classified as ORD had suggestive TB symptoms, but with negative microbiological *M.tb* diagnostics and were never initiated on TB treatment by the national TB control program.

### Sample Collection

For both the Norwegian and South African study participants, whole blood was collected by venepuncture into EDTA (Norway) or heparinized (South Africa) BD vacutainer tubes (BD Biosciences, Franklin Lakes, NJ, USA). After centrifugation (at 2,000 rpm for 10 min), plasma was harvested, aliquoted, and frozen at −80°C until use. Induced sputum samples and/or biopsies were obtained for acid-fast staining and culture by BACTEC 960 MGIT liquid culture media (BD Biosciences) or Lovenstein Jensen solid media. Positive MGIT cultures were examined for acid-fast bacilli using the Ziehl-Neelsen technique (to check for contamination).

### Ethical Considerations

The Norwegian participants were included in the ongoing cohort “*Prognostic Immunological markers in tuberculosis”* at the Department of Infectious Diseases, OUS, Norway (approved by Regional Ethics Committee, REK 2016/2123). Biological samples were stored in the biobank “*Research Biobank Infectious Diseases*” (REK nr.6.2008.173). South African participants enrolled into the *ScreenTB* study was approved by the Health Research Ethics Committee of the University of Stellenbosch (N16/05/070). All participants gave written informed consent before study inclusion. All methods were performed in accordance with the relevant guidelines and regulations.

### Multiplex Immunoassay

The 54 candidate TB diagnostic host markers were selected after literature searches (9, 10, 24, 28, 29) and evaluated in plasma specimens from all participants using the Luminex multiplex immunoassay platform. These markers are listed in Table 1. Samples were randomly assigned for testing on different assay plates, with the laboratory staff blinded to the clinical classification of study participants. All samples including the laboratory internal quality controls were diluted according to the recommendations of the manufacturers before analysis. The levels of the different biomarkers in the quality control reagents were within the expected ranges. Assays were performed on the Bio-Plex platform (Bio-Plex 200 and/or Magpix; Bio-Rad Laboratories, Hercules, USA) with the Bio-Plex Manager Software (version 6.1) used for bead acquisition and analysis of median fluorescent intensity, in an ISO15189:2007 accredited laboratory.

**Table 1:** Host markers evaluated in this study.

**Abbreviation**	**Full name**
**Reagent kits purchased from Merck Millipore, Billerica, Massachusetts, USA**	
CRP SAA SAP ApoA1 C1q C3b/iC3b CC3 CC4 CFB CFH	C-reactive protein Serum amyloid A Serum amyloid P component Apolipoprotein A1 Complement component 1q Complement component 3b Complement component 3 Complement component 4 Complement factor B Complement factor H
**Reagent kits purchased from R&D Systems, Minneapolis, Minnesota, USA**	
Anti-thrombin III ADAMTS13 TGF-α IFN-γ IP-10 TNF-α, TNF-β Ferritin Myoglobin PCT Pentraxin 3 CCL1/I-309 MIG/CXCL9 VEGF VEGFR3 GDF-15 NCAM TNFRII RANTES PDGF-BB MCP-1 MDC G-CSF ICAM-1 VCAM-1 sCD40L MPO MMP-(1, 2, 9) CCL18 MIP-1α, MIP-1β IL-(22, 1β, 12(p40),12(p70), 2, 8, 13) IL-1Ra IL-4Ra IL-2Ra IL-6Ra	Anti-thrombin III A disintegrin and metalloproteinase with a thrombospondin type 1 motif, member 13 Transforming growth factor alpha Interferon gamma IFN-γ-inducible protein Tumor necrosis factor-(alpha), beta Ferritin Myoglobin Procalcitonin Pentraxin 3 Chemokine (C-C motif) ligand 1 Monokine induced by gamma interferon Vascular endothelial growth factor Vascular endothelial growth factor receptor 3 Growth/differentiation factor 15 Neural cell adhesion molecule Tumor necrosis factor receptor 2 Regulated on activation, normal T cell expressed and secreted Platelet derived growth factor BB Monocyte chemoattractant protein 1 Macrophage derived chemokine Granulocyte colony stimulating factor Intercellular adhesion molecule 1 Vascular cell adhesion protein 1 Soluble CD40 ligand Myeloperoxidase Matrix metalloproteinase Chemokine (C-C motif) ligand 18 Macrophage inflammatory protein 1 (alpha), (beta) Interleukin Interleukin-1 receptor antagonist Interleukin-4 receptor alpha Interleukin-2 receptor alpha Interleukin-6 receptor alpha

### Statistical Analysis

Box-cox transformation and winsorization were performed in preparation for statistical analysis for analytes requiring transformation. Differences in the concentrations of host markers between the different groups were analyzed using the non-parametric Mann-Whitney U test. Mixed-effects linear models using the *lmer* package in the R were used to carry out univariate analyses for repeated measures (Baseline, Week 2, Month 2, and Month 6). The diagnostic abilities of host markers were assessed by receiver operator characteristics (ROC) curve analysis. Optimal cut-off values and associated sensitivity and specificity were determined based on the Youden's Index. The predictive abilities of combinations of host markers were investigated using general discriminant analysis (GDA). Depending on the size of the observations, data were randomly split into a training (70%) and test set (30%) whereby, models built on the training set were validated on the test set, otherwise, by leave-one-out cross-validation (that is, after each data point is removed, a model is built on the rest of the data and a prediction is made at that point and later tested on all the data). The best subset based on the Wilks lambda method was used in selecting analytes for the different biosignatures. *P* ≤ 0.05 were considered significant for differences between groups. The data were analyzed using Statistica (TIBCO Software Inc., CA, USA), Graphpad Prism version 8 (Graphpad Software Inc., CA, USA), and R programming language.

## Results

### Study Participants

Of a total of 185 included participants from both Norway and SA, 107 (57.8%) were diagnosed with TB and 78 (42.2%) with ORD. Among the Norwegian TB cases, 23 (68%)/21 (62%), and 13 (33%)/9 (23%) were confirmed TB (culture and/or PCR) in the PTB and EPTB cases, respectively. All 22 South African TB cases had PTB; 21 (95%) and 14 (64%) of whom were culture and smear-positive, respectively. The mean age of all TB cases was 36.8 ± 13.3 years, 9 (5%) were HIV infected and 69 (64%) were males. Participants with ORD had a mean age of 47.1 ± 14.6 years and 31 (40%) were males. An overview of the clinical and demographic characteristics of TB cases and ORD in the respective countries is shown in Table 2.

**Table 2:** Demographic and clinical characteristics of study participants.

**Characteristics**	**TB**		**ORD**	
	**Norway**	**South Africa**	**Norway**	**South Africa**
Participants (*N*)	85	22	19	59
Age, mean ± SD	36.7 ± 13.9	37.2 ± 11.2	60.6 ± 12.1	41.9 ± 13.8
Males, *n* (%)	55 (65)	14 (64)	7 (37)	24 (41)
HIV pos, *n* (%)	8 (9)	1 (5)	0 (0)	0 (0)
**Type of TB**, ***n*** **(%)**				
PTB EPTB PTB + EPTB	34 (40) 39 (46) 12 (14)	22 (100) / /	NA	NA
**Ethnicity**, ***n*** **(%)**				
Caucasian Asian African Colored (SA)	27 (32) 22 (26) 34 (40) /	/ / / 22 (100)	18 (95) 1 (5) / /	/ / 2 (3) 57 (97)

*Other Respiratory Diseases (ORD) in the Norwegian cohort consisted of lower respiratory infections, mostly confirmed as bacterial pneumonia by chest X-ray and routine blood cultures. As described in (12), the South African ORD group consisted of individuals with a range of other diagnoses including acute exacerbations of chronic obstructive pulmonary disease or asthma, upper and lower respiratory tract infections including viral and bacterial infections. Attempts to identify these organisms by bacterial or viral cultures were not made. TB, tuberculosis; ORD, other respiratory diseases; PTB, Pulmonary TB; EPTB, Extrapulmonary TB; SD, standard deviation; pos, positive*.

Plasma concentrations of I-309, MMP-1, MPO, PDGF-BB, RANTES, CRP, and Pentraxin3 show potential as TB diagnostic candidates irrespective of the study cohort.

#### Norwegian Cohort

The baseline concentrations of 15 of the 54 analytes investigated had significantly different levels in all TB patients (*n* = 85) compared to ORD patients (*n* = 19) (0.0465 < *P* < 0.0001) (Supplementary Table 1). There were significantly higher levels of I-309, MDC, VEGFR3, MMP-1, PDGF-BB, and RANTES in the TB patients compared to ORD, whereas the levels of CCL18, VCAM-1, GDF-15, MPO, pentraxin3, ferritin, myoglobin, CRP, and procalcitonin were significantly lower. The area under the ROC curve (AUC) was ≥ 0.70 for 10 of these markers namely, I-309, GDF-15, VEGFR3, MPO, MMP-1, Pentraxin3, PDGF-BB, RANTES, Ferritin, and CRP, whereas Myoglobin and Procalcitonin diagnosed TB with AUC ≥ 0.80 (Supplementary Figure 1). When only the individuals with pulmonary TB were compared to those with ORD, significant differences were observed for SAA, CRP, VEGFR3, RANTES, Pentraxin3, Ferritin, CCL18, MPO, GDF-15, MMP-1, PDGF-BB, Procalcitonin, MDC, Myoglobin, and VCAM-1. The diagnostic accuracies of these markers as ascertained by ROC curve analysis showed potential, with AUC ranging from 0.69 to 0.89 (Supplementary Table 2).

#### South African Cohort

The median baseline concentrations of 25 markers were significantly higher in TB patients than ORD patients namely; C3b/iC3b, IL-4Ra, C1q, procalcitonin, CFB, CCL18, GDF-15, VCAM-1, TNF-α, ferritin, MPO, SAA, CRP, IL-2Ra, IFN-γ, IP-10, PDGF-BB, VEGF, pentraxin3, MMP-1, RANTES, TNFRII, MIG, sCD40L, and I-309 (Supplementary Table 3). After ROC curve analysis, 13 of these biomarkers (MPO, SAA, CRP, IL-2Ra, IFN-γ, IP-10, PDGF-BB, VEGF, pentraxin3, MMP-1, RANTES, TNFRII, MIG discriminated between the TB and ORD groups with AUC ≥ 0.70 and sCD40L and I-309 were the most promising, with AUC ≥ 0.80 (Supplementary Figure 2).

#### Norwegian and South African Cohorts Combined

When data from all study participants were analyzed irrespective of the study site, the median concentrations of 21 of the 54 analytes were significantly different between the TB patients and those with ORD. The levels of SAA, VEGFR3, TNF-α, IL-2Ra, C1q, IL-12p70, MIG, TNFRII, C3b/iC3b, CC3, IP-10, I-309 were significantly higher in TB patients whereas, GDF-15, myoglobin, MMP-2, anti-thrombin III, IL-1Ra, MMP-9, and G-CSF levels were significantly higher in the ORD group (Supplementary Table 4). After ROC curve analysis, the AUC was ≥ 0.70 for CC3, IP-10, and I-309 (Supplementary Figure 3). When further univariate analysis was carried out in study participants from both cohorts excluding those with EPTB, the concentrations of C1q, CC3, C3b/iC3b, MIG, IL-12p70, TNFRII, VEGFR3, I-309, MIP-1a, IP-10, and G-CSF showed significant differences between the pulmonary TB and ORD groups (Supplementary Table 5).

**Figure 2:**
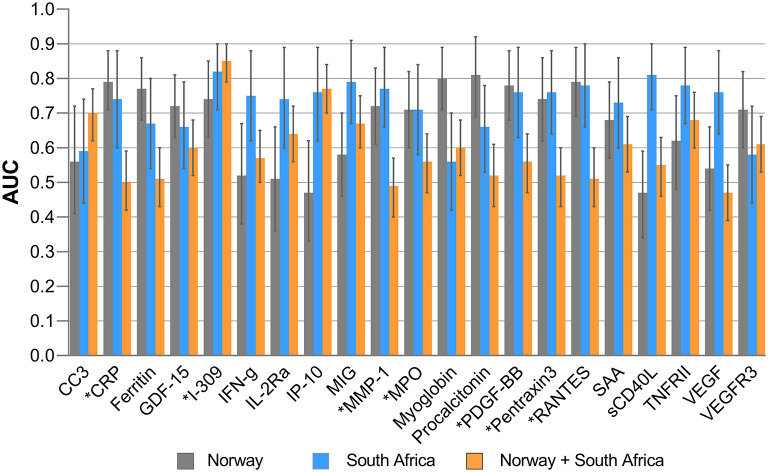
Areas under the receiver operator characteristics (ROC) curves of significant biomarkers. AUC for individual analytes with significant differences in Norway and South Africa and their performance when all study participants from both settings were merged. Error bars represent 95% confidence interval of AUC. ^*^Highlights markers with promising diagnostic accuracy in both Norway and South African cohorts.

Baseline concentrations of I-309, MPO, MMP-1, PDGF-BB, RANTES, CRP, and pentraxin3 thus showed diagnostic potential (AUC≥ 0.70) both in the Norwegian and South African cohorts. However, irrespective of the study site, I-309 was the most useful single marker that discriminated between TB and ORD (Figure 2). CCL18, CRP, GDF-15, ferritin, Procalcitonin, Pentraxin3, MPO, and VCAM-1 were highly expressed in patients with ORD from the Norwegian cohort in contrast to the South African cohort where these markers were higher in TB patients (Supplementary Figures 1, 2).

### Evaluation of Diagnostic Biosignatures in Tuberculosis

#### Norwegian Cohort

**Figure 3:**
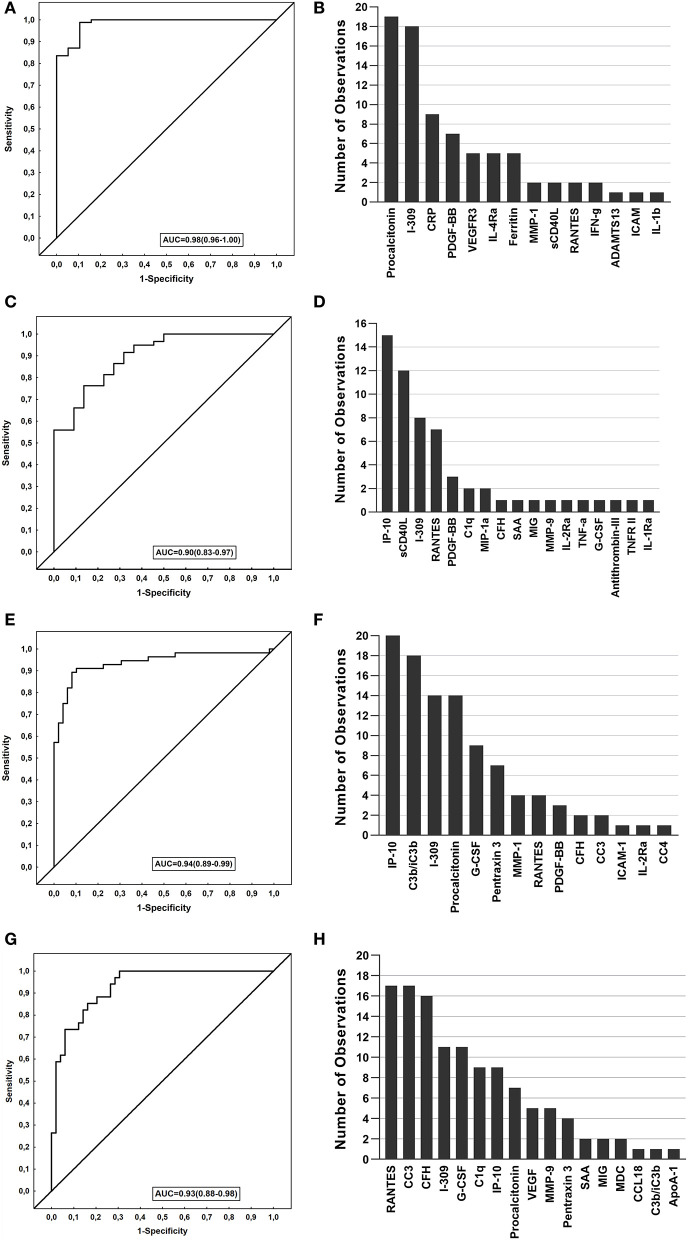
Performance of biosignatures in the diagnosis of TB disease. The Receiver operator characteristics (ROC) curves showing the accuracies of the biosignatures generated. The bar graphs show the number of times each analyte was included in the top 20 general discriminant analysis (GDA) models for diagnosing TB. **(A)** ROC curve of the most accurate 4-marker biosignature (I-309, procalcitonin, CRP, PDGF-BB) which diagnosed TB in the Norwegian cohort. **(B)** Frequency of analytes in the top 20 GDA models that classified TB cases from ORD in Norwegian patients. **(C)** ROC curve of the most accurate 3-marker biosignature (MMP-9, IP-10 and sCD40L) which diagnosed TB in the South African cohort. **(D)** Frequency of analytes in the top 20 GDA model that classified the TB cases from ORD in South African patients. **(E)** ROC curve of the most accurate 5-marker biosignature (G-CSF, C3b/iC3b, procalcitonin, IP-10, and PDGF-BB) in diagnosing TB in all study participants from both cohorts. **(F)** Frequency of analytes in the top 20 GDA model that classified the TB cases from ORD in all study participants from both cohorts. **(G)** ROC curve of the most accurate 6-marker biosignature (RANTES, G-CSF, C1q, CC3, CFH, IP-10) in diagnosing pulmonary TB in all study participants from both cohorts. **(H)** Frequency of analytes in the top 20 GDA models that classified pulmonary TB from ORD in all study participants from both cohorts.

We evaluated biosignatures in all Norwegian TB patients encompassing EPTB patients followed by analyses when only PTB was included and thereafter, assessed their performance on the South African cohort. When data obtained from the TB patients were analyzed by general discriminant analysis (GDA), optimal diagnosis of TB was achieved with a combination of four analytes. The most optimal biosignature was made up of 4-markers (I-309, procalcitonin, CRP, and PDGF-BB) which identified TB cases with an AUC of 0.98 (Figure 3A). After leave-one-out cross validation, the sensitivity of the 4-marker biosignature was 91.8% (95% CI, 83.8–96.8%) and specificity 89.5% (95% CI, 66.9–98.7%). The positive and negative predictive values (PPV and NPV) were 97.5% (95% CI, 91.3–99.3%) and 70.8% (54–83.4%), respectively. The frequency of markers in the top 20 most accurate 4-marker GDA models for the diagnosis of TB is shown in Figure 3B.

When the 4-marker biosignature was applied to South African study participants, with the latter being used as a validation cohort, this biosignature diagnosed TB with reduced sensitivity to 68.2% (95%CI, 45.1–86.1) and specificity of 91.5% (95% CI, 81.3–97.2) (Table 3). Moreover, when the signature was optimized to meet the minimal requirements of the World Health Organization (WHO) target product profile for a triage test for use in a high TB setting (sensitivity >90% and specificity >70%), the specificity obtained was 76% for the targeted sensitivity of 90%.

**Table 3:** Accuracy of the biosignatures identified in the present study in the diagnosis of active TB.

**Biosignature**	**AUC (95% CI)**	**Accuracy in training set**		**Accuracy in test set or after leave-one-out cross-validation**				**Performance according to WHO TPP**	
		**Sens (95%CI)**	**Spec (95%CI)**	**Sens (95%CI)**	**Spec (95%CI)**	**PPV (95%CI)**	**NPV (95%CI)**	**Sens (at Spec ≥70%)**	**Spec (at Sens ≥90%)**
**4-marker biosignature (I-309, procalcitonin, CRP, PDGF-BB) identified in the Norwegian cohort**									
	0.98 (0.96–1.00)	92.9% (85.3–97.4)	89.5% (66.9–98.7)	91.8% (83.8–96.6)	89.5% (66.9–98.7)	97.5% (91.3–99.3)	70.8% (54–83.4)	100	89
	**Performance of the 4-marker biosignature in the South African cohort**								
	0.90 (0.82–0.98)	68.2% (45.1–86.1)	93.2% (83.5–98.1)	68.2% (45.1–86.1)	91.5% (81.3–97.2)	75% (55.3–87.9)	88.5% (80.6–93.5)	91	76
**5- marker biosignature (C1q, procalcitonin, CRP, PDGF-BB, Ferritin) identified in Pulmonary TB from the Norwegian cohort**									
	1.00 (1.00–1.00)	100% (89.9–100)	100% (79.4–100)	100% (89.9–100)	100% (79.4–100)	100%	100%	100	100
	**Performance of the 5-marker biosignature in the South African cohort**								
	0.76 (0.65–0.88)	77.3% (54.6–92.2)	57.6% (44.1–70.4)	63.6% (40.7–82.8)	57.6% (44.1–70.4)	35.9 (26.6–46.4)	80.9 (70.1–88.5)	59	44
**3-marker biosignature (MMP-9, IP-10, sCD40L) identified in the South African cohort**									
	0.90 (0.83–0.97)	68.2% (45.1–86.1)	88.1% 77.1–95.1)	68.2% (45.1–86.1)	88.1% (77.1–95.1)	68.2% (50.3–82)	88.1% (80–93.2)	86	68
	**Performance of the 3-marker biosignature in the Norwegian cohort**								
	0.58 (0.44–0.72)	49.4% (38.4–60.5)	57.9% (33.5–79.7)	48.2% (37.3–59.3)	36.8% (16.3–61.6)	77.4% (69.4–83.7)	13.7% (7.9–22.9)	38	16
	**Performance of the 3-marker biosignature in Pulmonary TB from the Norwegian cohort**								
	0.50 (0.34–0.66)	38.2% (22.2–56.4)	68.4% (43.4–87.4)	29.4% (15.1–47.5)	31.6% (12.6–56.6)	43.5% (29.6–58.5)	20% (11.1–33.4)	47	0
**5-marker biosignature (G-CSF, C3b/iC3b, procalcitonin, IP-10, PDGF-BB) identified in the Norwegian and South African cohorts combined**									
	0.94 (0.89–0.99)	84.2% (72.1–92.5)	91.8% (80.4–97.7)	72.7% (49.8–82.3)	90.5% (69.6–98.8)	88.9% (67.2–96.8)	76% (61.2–86.4)	93	78
**6-marker biosignature (RANTES, G-CSF, C1q, CC3, CFH, IP-10) identified in Pulmonary TB from the Norwegian and South African cohorts combined**									
	0.93 (0.88–0.98)	82.4% (65.5–93.2)	83.7% (70.3–92.7)	66.7% (38.4–88.2)	81% (58.1–94.6)	71.4% (49.1–86.6)	77.3% (61.7–87.6)	91.2	73.5

*Biosignatures generated from data obtained in the Norwegian cohort (pulmonary TB + extrapulmonary TB), South African cohort (pulmonary TB) and when the cohorts (Norway and SA) were combined are shown. The Leave-one-out cross-validation method was applied to the Norwegian cohort (N = 104) and South African cohort (N = 81), whereas the training and test set method was used when both cohorts were combined (N = 185) to determine the predictive accuracy of biosignatures. The performance of biosignatures were also evaluated against the minimum sensitivity and specificity values of the minimum WHO target product profile (TPP) for a triage test. AUC, Area under the ROC curve; Sens, sensitivity; Spec, specificity; PPV, Positive Predictive Value; NPV, Negative Predictive Value; CI, Confidence Interval*.

When the analysis was performed only in study participants with PTB against ORD, optimal prediction of TB disease was achieved with the 5-marker signature made up of a combination of C1q, procalcitonin, CRP, PDGF-BB, and Ferritin. This 5-marker signature diagnosed TB with an accuracy of 100%, with sensitivity and specificity of 100% obtained after leave-one-out cross validation. The performance of the signature was reduced when applied to the South African cohort, with a sensitivity of 63.6% (95% CI, 40.7–82.8) and specificity 57.6% (95% CI, 44.1–70.4) (Table 3).

### South African Cohort

When data obtained from the South African participants were similarly fitted into GDA models, optimal prediction of TB was achieved with a combination of three markers. The most accurate 3-marker signature; MMP-9, IP-10 and sCD40L diagnosed TB with an AUC of 0.90 (95% CI, 0.83–0.97) (Figure 3C). After leave-one-out cross validation, the sensitivity of the biosignature was 68.2% and specificity was 88.1% (95% CI, 77.1–95.1%), with the PPV and NPV being 68.2% (95% CI, 50.3–82%) and 88.1% (95% CI, 80–93.2%), respectively (Table 3). When applied to the Norwegian cohort, the optimal 3 marker biosignature ascertained TB with reduced sensitivity and specificity of 48.2% (95% CI, 37.3–59.3) and 36.8% (95% CI, 16.3–61.6) respectively (Table 3). In the Norwegian study participants with PTB, an even reduced sensitivity of 29.4% (95% CI, 15.1–47.5) and specificity of 31.6% (95% CI, 12.6–56.6) was observed. Albeit the reduced diagnostic accuracy of the signature when applied to Norwegian study patients, the model ascertained TB with a specificity of 68% when optimizing for a higher sensitivity at ≥90%, and a sensitivity of 86% at a specificity ≥70%. The frequency of markers in the top 20 most accurate 3-marker GDA models for diagnosing TB is shown in Figure 3D.

#### Norwegian and South African Cohorts Combined

To identify the potentially most useful biosignature for diagnosing TB irrespective of the study cohort, participants from the two study sites were combined and randomly assigned into a training (70%) and test (30%) sets. A 5-marker signature comprising of G-CSF, C3b/iC3b, procalcitonin, IP-10 and PDGF-BB that was identified in the training sample set performed in the test set with a sensitivity of 72.7% (95% CI, 49.8–82.3%), specificity of 90.5% (95% CI, 69.6–98.8%), PPV of 88.9% (95% CI, 67.2–96.8%) and NPV of 76% (95% CI, 61.2–86.4%). The signature obtained a specificity of 78% when the target for sensitivity was set at 90%, therefore meeting the WHO TPP criteria for a triage TB test (Table 3). The most frequent markers in the top 20 most accurate 5-marker GDA models for discriminating between TB and ORD when participants were combined are shown in Figure 3F. When the patients with EPTB were excluded prior to analysis of the merged data (Norway and South-Africa), the most optimal TB diagnostic biosignature was comprised of six markers (RANTES, G-CSF, C1q, CC3, CFH, IP-10), and ascertained TB with an AUC of 0.93 (Table 3 and Figures 3G,H).

### Performance of Previously Identified African Signatures in the Norwegian Cohort

Finally, we evaluated the performance of previously reported host serum (CRP, SAA, IFN-γ, IP-10, CFH, ApoA-1, transthyretin) (9) and plasma (CRP, SAP, NCAM, ferritin, I-309/CCL-1, GDF-15) (10) biosignatures in the Norwegian cohort (Table 4). As transthyretin was not available for evaluation in this study, we assessed the performance of combinations between the other six markers from the Chegou et al. signature. Generally, these biosignatures performed well with good diagnostic accuracies (AUC 0.84 to 0.94) despite reduced sensitivity and specificity. The serum biosignature by Chegou et al. (9) identified TB disease with a sensitivity of 67.5% (95% CI, 56.3–77.4) and specificity of 64.7% (95% CI, 38.3–85.8) while the plasma biosignature by Jacobs et al. (10) obtained a sensitivity of 78.9% (95% CI, 69–86.8) and specificity of 89.5% (95% CI, 66.9–98.7). Similar diagnostic performance was obtained for these signatures when analyses were performed on the Norway and South Africa cohorts combined.

**Table 4:** Performance of previously identified African signatures in the diagnosis of TB disease in the Norwegian cohort and the combined Norwegian and South African cohort.

**Biosignature**	**AUC (95% CI)**	**Sens (95%CI)**	**Spec (95%CI)**	**Sens (95%CI)**	**Spec (95%CI)**	**PPV (95%CI)**	**NPV (95%CI)**
		**Accuracy in training set**		**Accuracy in test set or after leave-one-out cross-validation**			
**NORWEGIAN COHORT**							
*Chegou et al. (9) (CRP, SAA, IFN-γ, IP-10, CFH, ApoA-1)	0.84 (0.75–0.93)	69.9% (58.8–79.5)	88.2% (63.6–98.5)	67.5% (56.3–77.4)	64.7% (38.3–85.8)	90.3% (82.8–94.8)	30% (20.3–39.4)
Jacobs et al. (10) (CRP, SAP, NCAM-1, Ferritin, I-309, GDF-15)	0.94 (0.90–0.99)	81.2% (71.2–88.8)	94.7% (74–99.9)	78.8% (68.6–86.9)	89.5% (66.9–98.7)	97.1% (89.9–99.2)	48.6% (37.9–59.4)
**NORWEGIAN AND SOUTH AFRICAN COHORTS COMBINED**							
		**Training set (*****n*** **= 105;** ***n*** **= 56 TB**, ***n*** **= 49 ORD)**		**Test set (*****n*** **= 43;** ***n*** **= 22 TB**, ***n*** **= 21 ORD)**			
^*^Chegou et al. (9) (CRP, SAA, IFN-γ, IP-10, CFH, ApoA-1)	0.85 (0.77–0.92)	75% (61.6–85.6)	77.6% (63.4–88.2)	63.6% (40.7–82.8)	80.9% (58.1–94.5)	77.8% (57.8–89.9)	68% (54.1–79.3)
Jacobs et al. (10) (CRP, SAP, NCAM-1, Ferritin, I-309, GDF-15)	0.87 (0.80–0.94)	75% (61.6–85.6)	85.7% (72.8–94.1)	63.6% (40.7–82.8)	76.2% (52.8–91.7)	73.7% (55–86.5)	66.7% (52.3–78.5)

*Previously published biosignatures; a serum biosignature (9), and a plasma biosignature (10), were evaluated in the Norwegian cohort and in Norwegian and South African cohorts combined*.

* *One of the key biomarkers in the 7-marker serum biosignature (transthyretin) was unavailable, hence data shown is for performance of the remaining 6 analytes in the signature*.

*AUC, Area under the ROC curve; Sens, sensitivity; Spec, specificity; PPV, Positive predictive value; NPV, Negative predictive value; CI, Confidence Interval*.

### Changes in the Concentrations of Biomarkers During Treatment

**Figure 4:**
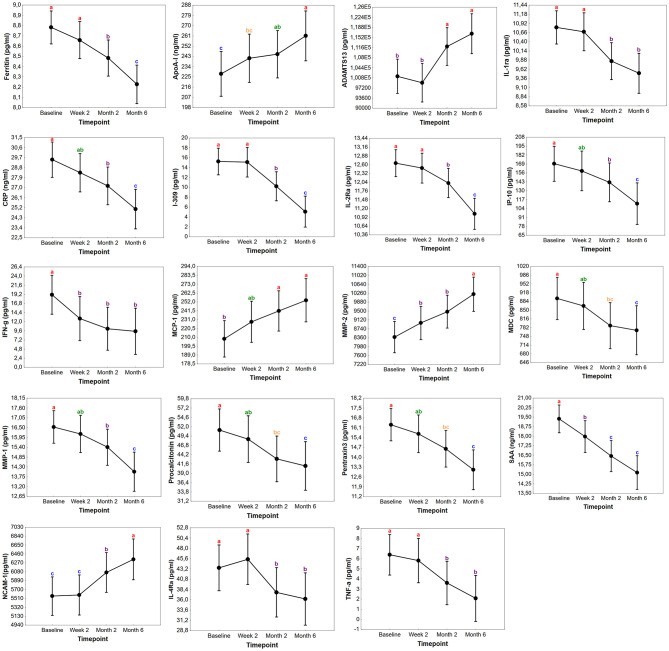
Changes in the concentrations of biomarkers in TB patients during treatment in Norway. Concentrations of plasma markers in samples of Norwegian TB patients with significant differences before the start of TB treatment (baseline), at week 2, month 2, and month 6. Data points in each graph represent the mean and the error bars indicate the 95% confidence intervals. The concentrations of ADAMTS13, MMP-2, NCAM-1, MCP-1, ferritin, I-309, IFN-γ, IL-1Ra, IL-2Ra, pentraxin3, procalcitonin, IP-10, MMP-1, TNF-α, IL-4Ra, MDC are expressed as pg/ml while ApoA-1, SAA, and CRP as ng/ml. The letters a-c indicates statistical significance where values with the same letter are not significantly different from each other.

To evaluate whether any of the biomarkers have the potential to be used for TB treatment monitoring, longitudinally collected plasma was analyzed in the Norwegian cohort. Out of the 85 TB cases 57 (67%), 62 (73%), and 49 (58%) provided specimens at week 2, months 2, and 6, respectively. The concentrations of 19 markers changed significantly in the course of TB treatment. There was a general decrease in the concentrations of CRP, ferritin, I-309, IFN-γ, IP-10, IL-1ra, IL-2Ra, IL-4Ra, MDC, MMP-1, pentraxin3, procalcitonin, TNF-α, and SAA from baseline to month 6, whereas ADAMTS13, MMP-2, MCP-1, ApoA-1, and NCAM-1 levels significantly increased in the course of treatment (Figure 4). Significant changes were already observed for IFN-γ, MMP-2, and SAA already at week 2 of treatment while levels of ADAMTS13, ApoA-1, CRP, ferritin, I-309, IL-1Ra, IL-4Ra, IP-10, MCP-1, MDC, MMP-1, NCAM-1, pentraxin3, procalcitonin and TNF-α became significantly changed first from month 2. The change in the concentrations of MIG, CCL18, TNFRII, IL-2, IL-12p70, and G-CSF only became significant at the end of treatment (month 6) compared to baseline levels. Finally, CFB, sCD40L, ICAM-1, IL-8, and VCAM-1 showed a decreasing trend during treatment with no overall significant changes (Supplementary Table 6).

## Discussion

We present data on the performance of plasma host biosignatures in patients from two different TB endemic settings to assess the usefulness of already identified promising biomarkers. An optimal 5-marker biosignature (G-CSF, C3b/iC3b, Procalcitonin, IP-10, PDGF-BB) was identified which diagnosed TB if no pre-conditions were set, with a sensitivity of 72.7% and specificity of 90.5% irrespective of geographical site. When optimized with sensitivity set at >90% (the minimum threshold set in the WHO TPPs for a triage TB test), the specificity of this 5-marker biosignature was 78%. When the specificity was fixed at >70%, the sensitivity of the biosignature was 93%. The performance of this biosignature therefore met the minimum requirements for a triage TB test in the current study. When evaluated as potential biomarkers for monitoring TB treatment response, the concentrations of 19 markers changed with treatment, thereby showing that they may be potential candidates for monitoring therapy responses.

The development of a rapid and simple non-sputum based immunodiagnostic tool will be ideal for the fight against TB in both high and low TB burden settings, most especially, in resource-limited settings. Validation of promising diagnostic host biomarkers for translation into novel point-of-care tests in multiple sites is thus of utmost importance. In our findings, I-309 (CCL1) was the most accurate single marker with diagnostic potential irrespective of the geographical site. I-309 is an inflammatory mediator, a member of the CC chemokine family, which stimulates the migration of human monocytes and whose expression is induced by Mtb and Toll-Like-Receptor (TLR) ligands expressed on macrophages (30). CCL18, CRP, GDF-15, ferritin, procalcitonin, pentraxin3, MPO, and VCAM-1 showed different response patterns in the different cohorts. They were highly expressed in patients with ORD in the Norwegian cohort and high among TB patients in the South African cohort. This could be a result of the differences in clinical settings in the two geographical sites. Whereas, in South Africa where the risk of TB is high, it is low in Norway for a patient admitted to the hospital with respiratory symptoms. Norwegian ORD patients were clinically diagnosed with lower respiratory infections, predominately bacterial pneumonia implying the presence of systemic inflammatory markers such as CRP, procalcitonin, and pentraxin3. The South-African ORD cohort was somewhat younger and more diverse with a range of respiratory tract infections which were not further investigated as previously reported by Chegou et al. (12). Furthermore, the differences between the highly expressed markers in study participants might be due to differences in geographical settings and ethnicity. Most of the patients in the Norwegian cohort were immigrants from different African and Asian countries as comparable to the South African cohort which was made up of mostly South African colored citizens. Some previous works showed that the inflammatory profile identified in TB was associated with ethnic variation in host genotype, (26, 31). Nevertheless, baseline concentrations of I-309, MMP-1, MPO, PDGF-BB, RANTES, CRP, pentraxin3 showed diagnostic potential for TB both in Norway, a low TB endemic setting, and in South Africa, a high TB burden area.

As observed in previous biomarker reports, the combination of different single markers performed better in diagnosing TB than individual markers. A 4-marker biosignature (I-309, CCL-1, procalcitonin, CRP, PDGF-BB) performed best in the Norwegian cohort, whereas a 3-marker biosignature (MMP-9, IP-10, sCD40L) was the most optimal in the South African cohort. When participants from both cohorts were combined, the 5-marker biosignature (G-CSF, C3b/iC3b, procalcitonin, IP-10, PDGF-BB) offered the best accuracy.

The WHO target profile (TPP) for a point-of-care non-sputum-based triage test capable of detecting people suspected of having TB recommends a diagnostic tool with a sensitivity > 90% and a specificity >70% (32). After optimizing the signatures identified in this study with these threshold values pre-specified, the specificities of the Norwegian 4-marker and the joint 5-marker signature fell within the accepted range (specificity >70%) for sensitivity > 90%. Thus, the diagnostic accuracy of these biosignatures identified in the present study meets the minimum WHO target product profiles for a triage test when benchmarked against these criteria. A test based on these signatures has the potential to be used as a good rule-out test for TB disease whilst awaiting further systematic screening in TB suspects most especially in high TB burden areas with limited resources. Bodily, further investigation is required on the performance of these biomarkers and signatures, including evaluation of the influence of HIV infection.

In defiance of different biosignatures identified in the present study, the Norwegian 4-marker signature showed potential as a rule-out test even when it was applied to South African participants after optimizing for higher sensitivity. Conjointly, the optimal 5-marker biosignature obtained when all sites were combined showed promise regardless of the different populations present in the joint cohort with heterogeneity in immune responses (pulmonary and EPTB TB patients combined). These findings were in contrast to the performance of the 3-marker signature identified in the South African cohort when assessed on the Norwegian cohort. We also observed a reduced sensitivity and specificity of previously published Africa-wide-derived 6-marker plasma and 7-marker serum signature (albeit, reduced to six markers because of the unavailability of one of the key biomarkers; transthyretin) in the Norwegian and combined cohorts. Although the reasons for the reduce performances is uncertain, there is a chance that biosignatures derived from studies designed differently, especially different levels of the health care settings may not validate in the respective cohorts. South Africa, a high burden area was represented by self-reporting individuals presenting with symptoms at a primary health care setting in contrast to hospitalized patients recruited in Norway, a high income and low TB burden setting. Other factors which might have influenced the differences observed in the current study could include differences in the types of both TB cases and individuals with ORD that were recruited at the different study sites, as well as the extent of disease in the patients, which was beyond the scope of the current study. In the Norwegian cohort, there were both PTB and EPTB cases as compared to the South African cohort that consisted only of definite PTB cases. Nonetheless, when the analysis was performed only on the PTB cases compared to the ORD group in the Norwegian cohort, an optimal signature of 5-markers (C1q, procalcitonin, CRP, PDGF-BB, ferritin) performed excellently with a sensitivity and specificity of 100%. The accuracy and performance of this signature were reduced when applied on the South African cohort while the identified South African 3-marker biosignature performed poorly in the new Norwegian data set (PTB vs. ORD). Although there might be differences in the immune response associated with PTB and EPTB, similar markers showing significant differences between TB and ORD were observed regardless of whether the EPTB patients were included or excluded during data analysis. That is, the markers that showed potential individually in discriminating between TB and ORD (C1q, CC3, C3b/iC3b, MIG, IL-12p70, TNFRII, VEGFR3, I-309, MIP-1a, IP-10, G-CSF in PTB vs. ORD) when Norwegian and South African study participants were combined were the same markers that showed potential when EPTB patients were excluded. That notwithstanding, a different 6-marker biosignature showed the most promise when only the PTB patients were compared to individuals with ORD (Norway and South Africa combined). Previous work by Fortún et al. highlighted no differences in biomarker concentrations between PTB and EPTB patients (33), whilst Ranaivomanana et al. reported differences in only TNF-α and VEGF after macrophage stimulation (34). Furthermore, blood transcriptional signatures reflecting immune response in PTB and EPTB patients were similar across sites of disease with varying degrees of responses correlating to the presence or absence of symptoms in another study (35). It is thus unclear whether geographical setting and patient recruitment strategy are the only contributing factors to the variation seen in the expression of biomarkers across sites. Still, all the promising biomarkers from the current study, are well-known proteins that have been widely investigated in TB, using different sample types (10, 24, 28). These markers in unison with frequently occurring markers in the top 20 GDA models may be considered strong candidate biomarkers for further investigations in multi-centre studies and point-of-care TB test development.

The concentration of several biomarkers changed significantly during TB treatment when evaluated in the Norwegian cohort. These markers could thus be useful as potential markers for monitoring TB treatment. The changes in concentrations after treatment initiation could be reflective of host immune function restoration due to a reduction in bacterial load. Our observation of increasing levels of ApoA-1, MMP-2 and MCP-1 is in agreement with previous reports (10, 36, 37). Amongst markers with a significant decrease during treatment were acute phase proteins (CRP, SAA, pentraxin3, procalcitonin and ferritin) as well as the pro-inflammatory markers IP-10, IFN-γ, TNF-α, and MMP-1 which are in accordance with several other works reporting their potential in evaluating TB treatment and as markers of disease severity and bacterial burden (10, 36, 38–42). Withal, host biomarkers have also shown potential in predicting month 2 culture status and poor TB treatment outcomes (relapse and failure), but assessment of the predictive accuracy of the biomarkers for different treatment outcomes was outside the scope of this study.

A particular strength of this study is the inclusion of study participants from different settings, which allowed us to test the robustness of previously published biomarkers in the diagnosis of TB in patients recruited using different protocols across different geographical settings. Investigations were carried out in a data set with a lot of heterogenicity between the study cohorts in terms of the ORD group and type of TB cases. Our study provides evidence that some of the biomarkers investigated may be strong, robust candidates for a globally relevant test. We also evaluated the usefulness of the biomarkers as tools for monitoring TB treatment responses in clinically responded patients at that time point, although we could not evaluate treatment outcome due to the small size of month 2 culture positive samples. Additionally, we were unable to assess the influence of HIV infection on the performance of the biomarkers due to the low proportion (5%) of the HIV infected study participants. We acknowledge that the proportion of HIV positive study participants particularly in the South African cohort was not representative of what is estimated for Sub-Saharan African countries. This bias was introduced by the random selection of study participants from our biobank, with the clinical information of participants only known during data analysis. That notwithstanding, previous work carried out in South Africa showed that the identified biosignatures performed well irrespective of HIV infection (9, 10, 13). However, well-designed studies in which the performance of the signatures is assessed in HIV positive patients that are stratified according to CD4 cell counts and viral loads are required. As the cohorts used in the present study were recruited using different strategies (hospitalized patients recruited in a low TB endemic setting vs. self-reported patients presenting with symptoms requiring investigation for TB at a high burden setting), not using the same protocols for recruitment of study participants may be seen as a limitation. The lack of sample size calculations prior to the start of the study is a limitation. However, the number of study participants employed in the study is similar to the numbers used in other previous biomarker-based work. Further multi-center confirmatory studies including people recruited in both high and low TB endemic settings are thus required. It may be necessary to standardize the study protocols so that bias due to different study designs does not affect study findings. However, it is important that such future studies enroll participants that are relevant to the different clinical settings, so that findings are clinically relevant. The biomarkers that performed well in the study may be considered as strong candidates for future evaluation and consideration for globally relevant point-of-care tests.

In conclusion, among 54 potential TB biomarkers evaluated in this study, we identified strong individual candidate biomarkers and a 5-marker plasma protein biosignature (G-CSF, C3b/iC3b, procalcitonin, IP-10, PDGF-BB) which showed potential in diagnosing TB regardless of the endemic setting. The concentrations of some of the markers also changed significantly during TB treatment, suggesting their potential utility as biomarkers for monitoring response to TB treatment. Our data highlights the importance of validating host immunological biomarkers in different geographical and ethnic settings, in the global search for non-sputum-based biomarkers for point-of-care diagnosis of active TB.

## Data Availability Statement

The raw data supporting the conclusions of this article will be made available by the authors, without undue reservation.

## Ethics Statement

The studies involving human participants were reviewed and approved by Regional Ethics Committee REK 2016/2123) at the Department of Infectious Diseases, OUS, Research Biobank Infectious Disease (REK nr.6.2008.173), and the Health Research Ethics Committee of the University of Stellenbosch (N16/05/070). The patients/participants provided their written informed consent to participate in this study.

## Author Contributions

NC and AD-R had the idea for and designed the study. NC, GW, and AD-R set up the clinical cohorts at the respective hospitals. KT, SJ, AD-R, GW, and NC included patients and collected data. CS and BC performed the multiplex analyses. BC and MK did the statistical analyses. BC, NC, and AD-R interpreted the data and drafted the paper. All authors critically reviewed the manuscript for important intellectual content and gave final approval for the version to be published. All authors agree to be accountable for all aspects of the work in ensuring that questions related to the accuracy or integrity of any part of the work are appropriately investigated and resolved.

## Conflict of Interest

The authors declare that the research was conducted in the absence of any commercial or financial relationships that could be construed as a potential conflict of interest.

## The ScreenTB Consortium

Stellenbosch University, South Africa: Gerhard Walzl, Novel N. Chegou, Petri Ahlers, Stephanus T. Malherbe, Gian van der Spuy, Ilana van Rensburg, Hygon Mutavhatsindi, Portia Manngo, Kim Stanley, Andriette Hiemstra, Shirley McAnda. Medical Research Council Gambia at LSHTM: Jayne Sutherland, Joseph Mendy, Awa Gindeh, Georgetta Mbayo, Ebrima Trawally, Olumuyiwa Owolabi Makerere University, Uganda: Harriet Mayanja-Kizza, Mary Nsereko, Anna-Rita Namuganga, Saudah Nambiru Kizito. Armauer Hansen Research Institute, Addis Ababa, Ethiopia: Adane Mihret, Sosina Ayalew, Rawleigh Howe, Azab Tarekegne, Bamlak Tessema. University of Namibia, Namibia: Emmanuel Nepolo, Joseph Sheehama, Gunar Gunther, Azaria Diergaardt, Uapa Pazvakavambwa. London School of Hygiene and Tropical Medicine, UK: Hazel Dockrell. Leiden University Medical Centre, Netherlands: Tom Ottenhoff, Elisa Tjon Kon Fat, Shannon Herdigein, Paul Corstjens, Annemieke Geluk.
