# Supplementary material for: A Plasma 5-Marker Host Biosignature Identifies Tuberculosis in High and Low Endemic Countries
Source: Front Immunol. 2021 Feb 24;12:608846. doi: 10.3389/fimmu.2021.608846 (PMC7958880; doi:10.3389/fimmu.2021.608846)
Supplement: Supplementary file 1 [file Data_Sheet_1.docx]

# Supplementary Tables

**Table 1: Median levels (and inter-quartile ranges in parenthesis) of host biomarkers detected in all Norwegian TB patients (pulmonary and extrapulmonary TB) and individuals with other respiratory diseases (ORD) and their diagnostic accuracies for TB disease**. Data obtained for participants recruited in Norway only is shown in the table. Only markers (15) with significant differences (p ≤ 0.05) between groups by the Mann Whitney U test are shown. Optimal cut-off values and associated sensitivity and specificity were determined based on the Youden’s index. All concentrations are in pg/ml with the exemption of CRP in ng/ml. Sens: Sensitivity, Spec: Specificity

| **Biomarker** | **TB**  **(N=85)**  **(IQR)** | **ORD**  **(N= 19) (IQR)** | **P value** | **AUC (95% CI)** | **Cut off value** | **Sens % (95% CI)** | **Spec % (95% CI)** |
| --- | --- | --- | --- | --- | --- | --- | --- |
| **CCL18** | 41377  (25867-  53843) | 48770 (38454-71117) | 0.0465 | 0.65  (0.52–0.77) | > 42523 | 74 (51 – 88) | 53 (42 – 63) |
| **MDC** | 832,7  (635,4-  1039) | 647.4 (465.1-798.1) | 0.0298 | 0.66  (0.51–0.81) | < 698.4 | 63 (41 – 81) | 69 (59 – 78) |
| **VCAM-1** | 826429  (626828-  1084050) | 1424900 (725699-1967700) | 0.0108 | 0.69  (0.54–0.83) | > 1259150 | 63 (41 – 81) | 82 (73 – 89) |
| **VEGFR3** | 729,4  (402,1-  1121) | 434.6 (293.4-634.2) | 0.0037 | **0.71**  **(0.60-0.82)** | < 634.7 | 79 (57 – 91) | 58 (47 – 68) |
| **MPO** | 35312  (25881-  48995) | 47298 (40091-63064) | 0.0035 | **0.71**  **(0.60–0.82)** | > 40010 | 79 (57 - 91) | 64 (52 – 73) |
| **GDF-15** | 360,5  (277,2-  834,5) | 701.0 (551.3-874.2) | 0.0024 | **0.72**  **(0.63–0.81)** | > 532.4 | 84 (62 -94) | 64 (53 - 73) |
| **MMP-1** | 1137  (578,4-  2342) | 512.2 (338.0-921.8) | 0.0019 | **0.72**  **(0.61–0.83)** | < 663.6 | 68 (46 – 85) | 7 (60 – 79) |
| **I-309** | 10,47  (4,950-  21,86) | 4.950 (0.000-7.580) | 0.0009 | **0.74**  **(0.63–0.85)** | < 5.935 | 58 (36-77) | 68 (57 - 77) |
| **Pentraxin 3** | 522,1  (362,3-  1012) | 1463 (513.2-2075) | 0.0008 | **0.74**  **(0.62–0.86)** | > 889.6 | 74 (51 – 88) | 72 (61 – 80) |
| **Ferritin** | 80125  (33319-  233565) | 246706 (157998-407602) | 0.0002 | **0.77**  **(0.68–0.86)** | > 138869 | 84 (62 – 94) | 68 (57 – 77) |
| **PDGF-BB** | 234,1  (7,525-  723,6) | 0.000 (0.000-104.8) | <0.0001 | **0.78**  **(0.68–0.88)** | < 440.4 | 100  (83 –100) | 38 (28 – 48) |
| **RANTES** | 7763  (2957-  18122) | 1013 (282.6-6053) | <0.0001 | **0.79**  **(0.69–0.89)** | < 2108 | 63 (41 – 81) | 84 (74 – 90) |
| **CRP** | 393774  (136572-  733872) | 905124 (583363-1172000) | <0.0001 | **0.79**  **(0.71–0.88)** | > 542706 | 84 (62 – 94) | 67 (57 – 76) |
| **Myoglobin** | 2551  (1897-  3864) | 4504 (4063-6027) | <0.0001 | **0.80**  **(0.71–0.89)** | > 3863 | 84 (62 – 94) | 75 (65 – 83) |
| **Procalcitonin** | 45,68  (28,46-  65,70) | 135.6 (57.87-224.6) | <0.0001 | **0.81**  **(0.69–0.92)** | > 84.27 | 74 (51 – 88) | 86 (77 – 92) |

**Table 2: Median levels (and inter-quartile ranges in parenthesis) of host biomarkers detected in Norwegian pulmonary TB patients and individuals with other respiratory diseases (ORD) and their diagnostic accuracies for TB disease**. Data obtained from study participants recruited in Norway only are shown. Only markers (15) with significant differences (p ≤ 0.05) between groups by the Mann Whitney U test are shown. Optimal cut-off values and associated sensitivity and specificity were determined based on the Youden’s index. All concentrations are in pg/ml with the exemption of SAA and CRP in ng/ml. Sens: Sensitivity, Spec: Specificity

| **Biomarker** | **PTB**  **(N=34) (IQR)** | **ORD**  **(N= 19) (IQR)** | **P value** | **AUC (95% CI)** | **Cut off value** | **Sens % (95% CI)** | **Spec % (95% CI)** |
| --- | --- | --- | --- | --- | --- | --- | --- |
| **MDC** | 867,4  (677,6-  1071) | 647.4 (465.1-798.1) | 0.0270 | 0.68  (0.52-0.84) | < 763.0 | 74 (51-88) | 65 (48-79) |
| **CCL18** | 36975  (23043-  53828) | 48770 (38454-71117) | 0.0221 | 0.69  (0.55-0.83) | > 41596 | 74 (51-88) | 62 (45-76) |
| **MMP-1** | 961,6  (508,8-  1926) | 512.2 (338.0-921.8) | 0.0235 | 0.69  (0.54-0.83) | < 615.9 | 63 (41-81) | 71 (54-83) |
| **VEGFR3** | 708,1  (396,5-  1075) | 434.6 (293.4-634.2) | 0.0088 | **0.72**  **(0.58-0.86)** | < 490.6 | 63 (41-81) | 68 (51-81) |
| **GDF-15** | 405,8  (290,8-  833,9) | 701.0 (551.3-874.2) | 0.0083 | **0.72**  **(0.58-0.85)** | > 532.4 | 84 (62-94) | 62 (45-76) |
| **VCAM-1** | 832845  (621182-  999406) | 1424900 (725699-1967700) | 0.0055 | **0.73**  **(0.57-0.89)** | > 853440 | 74 (51-88) | 56 (39-71) |
| **SAA** | 46690  (9221-  1062000) | 1062000  (1062000-  1062000) | 0.0005 | **0.75**  **(0.62-0.88)** | > 586485 | 89 (69-98) | 56 (39-71) |
| **RANTES** | 6335  (2131-  17687) | 1013 (282.6-6053) | 0.0017 | **0.76**  **(0.62-0.89)** | < 2113 | 63 (41-81) | 79 (63-90) |
| **MPO** | 32435  (24140-  45359) | 47298 (40091-63064) | 0.0018 | **0.76**  **(0.62-0.89)** | > 36441 | 84 (62-94) | 62 (45-76) |
| **Ferritin** | 79767  (38011-  210898) | 246706 (157998-407602) | 0.0007 | **0.78**  **(0.65-0.90)** | > 131130 | 84 (62-94) | 71 (54-83) |
| **PDGF-BB** | 250,7  (72,70-  629,3) | 0.000 (0.000-104.8) | 0.0003 | **0.78**  **(0.66-0.91)** | < 118.7 | 84 (62-94) | 74 (57-85) |
| **Myoglobin** | 2610  (1857-  3907) | 4504 (4063-6027) | 0.0003 | **0.79**  **(0.67-0.91)** | > 3863 | 84 (62-94) | 76 (60-87) |
| **Pentraxin 3** | 503,9  (336,3-  750,4) | 1463 (513.2-2075) | 0.0003 | **0.79**  **(0.66-0.93)** | > 830.0 | 74 (51-88) | 79 (63-90) |
| **Procalcitonin** | 46,17  (30,90-  62,24) | 135.6 (57.87-224.6) | <0.0001 | **0.84**  **(0.72-0.97)** | > 79.48 | 74 (51-88) | 94 (81-99) |
| **CRP** | 353083  (64496-  518917) | 905124 (583363-1172000) | <0.0001 | **0.89**  **(0.80-0.98)** | > 529273 | 95 (75-100) | 82 (66-92) |

**Table 3: Median levels (and inter-quartile ranges in parenthesis) of host biomarkers detected in South African** **pulmonary TB patients and individuals with other respiratory diseases (ORD) and their diagnostic accuracies for TB disease**. Data obtained from study participants recruited in South African only are shown. Only markers (25) with significant differences (p ≤ 0.05) between groups by the Mann Whitney U test are shown. Optimal cut-off values and associated sensitivity and specificity were determined based on the Youden’s index. All concentrations are in pg/ml with the exemption of C1q, CFB, SAA and CRP in ng/ml. Sens: Sensitivity, Spec: Specificity

| **Biomarker** | **TB**  **(N=22)**  **(IQR)** | **ORD**  **(N= 59)**  **(IQR)** | **P value** | **AUC**  **(95% CI)** | **Cut off value** | **Sens % (95% CI)** | **Spec % (95% CI)** |
| --- | --- | --- | --- | --- | --- | --- | --- |
| **C3b/iC3b** | 35095  (0,000-  134818) | 0,000  (0,000-  35095) | 0.0329 | 0.64  (0.50-0.77) | < 529.5 | 66 (53-77) | 59 (39-77) |
| **IL-4Ra** | 40.89  (33.23-52.64) | 34.49 (30.99-41.59) | 0.0360 | 0.65  (0.51-0.80) | < 36.69 | 63 (50-74) | 68 (47-84) |
| **C1q** | 120453  (107450-142181) | 109940(100881-126371) | 0.0289 | 0.66  (0.52-0.79) | < 112409 | 56 (43-68) | 73 (52-87) |
| **Procalcitonin** | 57.92  (42.07-82.99) | 36.91  (23.12-  66.03) | 0.0298 | 0.66  (0.53-0.78) | < 50.55 | 61 (48-72) | 73 (52-87) |
| **CFB** | 390912  (336835-433379) | 347878  (279274-  415672) | 0.0282 | 0.66  (0.53-0.79) | < 382108 | 69 (57-80) | 64 (43-80) |
| **CCL18** | 58653  (39651-  105254) | 44842  (25577-  65905) | 0.0276 | 0.66  (0.53-0.79) | < 57082 | 71 (59-81) | 59 (39 – 77) |
| **GDF-15** | 772.1  (653.1-1182) | 610.2  (382.5-  994.2) | 0.0231 | 0.66  (0.54-0.79) | < 721.3 | 63 (50-74) | 73 (52-87) |
| **VCAM-1** | 897495  (709655-  1232975) | 766227  (568086-  1023300) | 0.0219 | 0.67  (0.53-0.80) | < 886055 | 66 (53-77) | 54 (35-73) |
| **TNF-a** | 3.040  (0.000-  7.975) | 0.000  (0.000-  2.030) | 0.0106 | 0.67  (0.53-0.81) | < 0.6400 | 58 (45-69) | 68 (47-84) |
| **Ferritin** | 157891  (78503-  377407) | 84327  (30439-  198380) | 0.0185 | 0.67  (0.54-0.80 | < 96242 | 54 (42-66) | 73 (52-87) |
| **MPO** | 65087  (40842-  97926) | 45154  (28910-  60899) | 0.0039 | **0.71**  **(0.58-0.84)** | < 55926 | 73 (60-83) | 68 (47-84) |
| **SAA** | 1062000  (15194-  1062000) | 19410  (7959-  105813) | 0.0010 | **0.73**  **(0.60-0.86)** | < 87621 | 75 (62-84) | 73 (52-87) |
| **CRP** | 1169750  (375810-  1379275) | 327895  (82637-  798207) | 0,0006 | **0.74**  **(0.60-0.88)** | < 822185 | 78 (66-87) | 64 (43-80) |
| **IL-2Ra** | 1242  (691.8-2000) | 753.7  (611.7-  928.6) | 0.0006 | **0.74**  **(0.60-0.89)** | < 1024 | 88 (77-94) | 64 (43-80) |
| **IFN-g** | 33.51  (4.598-64.34) | 0.000  (0.000-  16.14) | 0.0001 | **0.75**  **(0.62-0.88)** | < 11.14 | 64 (52-75) | 73 (52-87) |
| **IP-10** | 89.82  (36.14-205.5) | 39.39  (28.34-  66.31) | 0.0003 | **0.76**  **(0.62-0.89)** | < 53.26 | 64 (52-75) | 73 (52-87) |
| **PDGF-BB** | 4300  (2427-8818) | 1591  (415.3-  3088) | 0.0003 | **0.76**  **(0.63-0.89)** | < 2181 | 64 (52-75) | 82 (61-93) |
| **VEGF** | 61.94  (32.21-99.98) | 30.57  (23.77-  42.26) | 0.0002 | **0.76**  **(0.64-0.88)** | < 40.66 | 73 (60-83) | 73 (52-87) |
| **Pentraxin3** | 936.6  (633.9-2171) | 495.5  (315.0-  743.7) | 0.0003 | **0.76**  **(0.64-0.88)** | < 657.2 | 69 (57-80) | 77 (57-90) |
| **MMP-1** | 4672  (3185-11892) | 1764  (1044-  3192) | <0.0001 | **0.77**  **(0.66-0.89)** | < 3377 | 78 (66-87) | 77 (57-90) |
| **RANTES** | 74702  (37906-  139488) | 25448  (12210-  48482) | <0.0001 | **0.78**  **(0.66-0.90)** | < 37391 | 66 (53-77) | 82 (61-93) |
| **TNF RII** | 2982  (2302-4793) | 1984  (1557-  2489) | <0.0001 | **0.78**  **(0.67-0.89)** | < 2280 | 73 (60-82) | 82 (61-93) |
| **MIG** | 1079  (572.4-3067) | 572.4  (0.000-  572.4) | <0.0001 | **0.79**  **(0.67-0.91)** | < 708.2 | 85 (73-92) | 64 (43-80) |
| **sCD40L** | 1618  (1376-4421) | 939.3  (612.4-  1457) | <0.0001 | **0.81**  **(0.71-0.90)** | < 1374 | 71 (59-81) | 77 (56-90) |
| **CCL1/I-309** | 7.460 (3.230-35.55) | 0.000(0.000-1.730) | <0.0001 | **0.82**  **(0.70-0.94)** | < 4.490 | 86 (75-93) | 73 (52-87) |

**Table 4: Median levels (and inter-quartile ranges in parenthesis) of host biomarkers detected in Norwegian and South-African TB patients combined (pulmonary and extrapulmonary TB) and individuals with other respiratory diseases (ORD) and their diagnostic accuracies for TB disease**. Data shown was obtained after measurements in both sets of study participants (Norway and South Africa) were jointly analyzed. Only markers (21) with significant differences (p ≤ 0.05) between groups by the Mann Whitney U test are shown. Optimal cut-off values and associated sensitivity and specificity were determined based on the Youden’s index. All concentrations are in pg/ml with the exemption of C1q, C3b/iC3b, CC3, SAA and CRP in ng/ml. Sens: Sensitivity, Spec: Specificity.

| **Biomarker** | **TB**  **(N=107)**  **(IQR)** | **ORD (N=78)**  **(IQR)** | **P value** | **AUC**  **(95% CI)** | **Cut off value** | **Sens % (95% CI)** | **Spec% (95% CI)** |
| --- | --- | --- | --- | --- | --- | --- | --- |
| **GDF-15** | 461,8  (293,5-906,3) | 680.0  (423.5 -  922.2) | 0.0186 | 0.60  (0.52 -0.68) | > 532.8 | 63 (52 –73) | 55 (46 – 64) |
| **Myoglobin** | 2706  (2003-4063) | 3564  (2609 -  4693) | 0.0194 | 0.60  (0.52 -0.68) | > 2951 | 67 (56– 76) | 59 (49 – 68) |
| **MMP-2** | 244293  (153623-  311399) | 302078  (204167 -  382224) | 0.0184 | 0.60  (0.52 -0.69) | > 271032 | 62 (50– 72) | 60 (50 -67) |
| **Antithrombin-III** | 3400200  (2525200-  4561300) | 4040800  (3106825 -4953100) | 0.0270 | 0.60  (0.51 -0.68) | > 3380900 | 71 (60– 79) | 50 (40 – 59) |
| **IL-1Ra** | 542,1  (394,6-1059) | 737.3  (514.4 -  1041) | 0.0270 | 0.60  (0.51- 0.68) | > 644.6 | 58 (47– 68) | 64 (55 – 73) |
| **SAA** | 1062000  (19473-  1062000) | 44139  (9299 -  1062000) | 0.0070 | 0.61  (0.53- 0.69) | < 160272 | 62 (50– 72) | 61 (52 – 69) |
| **VEGFR3** | 735,6  (425,2-1116) | 562.5  (363.7 -  861.7) | 0.0096 | 0.61  (0.53 -0.69) | < 658.7 | 62 (50– 71) | 55 (46 – 64) |
| **IL-2** | 0.000  (0.000 -27.34) | 0.000  (0.000 -  0.000) | 0.0016 | 0.61  (0.53 -0.69) | < 1.690 | 83 (74– 90) | 39 (31 – 49) |
| **TNF-a** | 2,030  (0,000-4,990) | 0.000  (0.000 -  3.040) | 0.0040 | 0.62  (0.54 -0.70) | < 1.035 | 64 (53– 74) | 60 (50 – 69) |
| **IL-2Ra** | 1009  (675,0-1503) | 782.5  (604.8 -  1013) | 0.0011 | 0.64  (0.56 -0.72) | < 978.0 | 72 (61– 81) | 56 (46 – 65) |
| **MMP-9** | 41794  (24341-  80947) | 70881  (36022 -  113819) | 0.0044 | 0.62  (0.54 -0.71) | > 57334 | 62 (50– 72) | 65 (56 – 74) |
| **C1q** | 120952  (103346-  145940) | 110980  (97792 -  125659) | 0.0073 | 0.63  (0.54-0.71) | < 112134 | 54 (43– 65) | 64 (53- 74) |
| **G-CSF** | 151,1  (121,2-178,2) | 179.1  (144.6 -  203.8) | 0.0001 | 0.66  (0.58 -0.74) | > 159.0 | 67 (55– 76) | 63 (53 – 71) |
| **IL-12p70** | 8,020  (0,000-20,45) | 0.000  (0.000 -  11.16) | <0.0001 | 0.67  (0.59- 0.75) | < 0.4650 | 63 (52– 73) | 69 (60 - 77) |
| **MIG** | 870,3  (0,000-1723) | 550.3  (0.000 -  572.4) | <0.0001 | 0.67  (0.60 -0.75) | < 587.8 | 81 (71– 88) | 59 (49 – 68) |
| **MIP-1a** | 131,1  (0,000-180,6) | 0.000  (0.000 -  82.84) | <0.0001 | 0.67  (0.60 -0.75) | < 84.63 | 77 (66– 85) | 56 (47 – 65) |
| **TNF RII** | 3280  (2470-4763) | 2220  (1759 -  3542) | <0.0001 | 0.68  (0.60 -0.76) | < 2378 | 59 (48– 69) | 79 (71 – 86) |
| **C3b/iC3b** | 138630  (0,000-  392695) | 0.000  (0.000 -  87807) | <0.0001 | 0.68  (0.61 -0.76) | < 1772 | 62 (51– 72) | 69 (60 – 77) |
| **CC3** | 146358  (91515-  258872) | 83126  (72474 -  123333) | <0.0001 | **0.70**  **(0.62-0.77)** | < 101004 | 64 (52– 73) | 73 (63 – 80) |
| **IP-10** | 117,0  (68,29-217,6) | 50.95  (30.07 -  84.08) | <0.0001 | **0.77**  **(0.70 -0.84)** | < 69.96 | 71 (60– 79) | 75 (66 – 82) |
| **CCL1/I-309** | 9,110  (4,740-22,75) | 0.000  (0.000 -  4.330) | <0.0001 | **0.85**  **(0.79 -0.90)** | < 3.715 | 74 (64– 83) | 81 (73 – 87) |

**Table 5: Median levels (and inter-quartile ranges in parenthesis) of host biomarkers detected in Norwegian and South-African pulmonary TB patients combined and individuals with other respiratory diseases (ORD) and their diagnostic accuracies for TB disease.** Data shown was obtained after measurements in both sets of study participants (Norway and South Africa) jointly analyzed excluding EPTB cases. Only markers (11) with significant differences (p ≤ 0.05) between groups by the Mann Whitney U test are shown. Optimal cut-off values and associated sensitivity and specificity were determined based on the Youden’s index. All concentrations are in pg/ml with the exemption of C1q, C3b/iC3b, and CC3 in ng/ml. Sens: Sensitivity, Spec: Specificity.

| **Biomarker** | **PTB**  **(N=56)**  **(IQR)** | **ORD (N=78)**  **(IQR)** | **P value** | **AUC**  **(95% CI)** | **Cut off value** | **Sens % (95% CI)** | **Spec% (95% CI)** |
| --- | --- | --- | --- | --- | --- | --- | --- |
| **VEGFR3** | 732.5  (428,1-  1069) | 562.5  (363.7-  861.7) | 0.0351 | 0.61  (0.51-0.71) | < 634.6 | 56  (45-67) | 61  (48-72) |
| **MIP-1a** | 0.000  (0.000-  164.7) | 0.000  (0.000-  82.84) | 0.0148 | 0.61  (0.51-0.70 | < 26.06 | 72  (61-81) | 48  (36-61) |
| **IL-12p70** | 8.020  (0.000-  13.88) | 0.000  (0.000-  11.16) | 0.0125 | 0.62  (0.52-0.71) | < 1.750 | 63  (52-72) | 63  (49-74) |
| **G-CSF** | 155.2  (123.3-  193.1) | 179.1  (144.6-  203.8) | 0.0131 | 0.63  (0.53-0.72) | > 174.4 | 55  (44-67) | 68  (55-79) |
| **C1q** | 123528  (103639-  150328) | 110980  (97792-  125659) | 0.0070 | 0.64  (0.54-0.75) | < 113974 | 63  (51-73) | 62  (48-74) |
| **C3b/iC3b** | 111035  (0.000-  500816) | 0.000  (0.000-  87807) | 0.0003 | 0.67  (0.58-0.77) | < 1772 | 62  (51-72) | 66  (53-77) |
| **CC3** | 126683  (87524-  383693) | 83126  (72474-  123333) | 0.0005 | 0.68  (0.58-0.77) | < 101004 | 64  (52-73) | 68  (55-79) |
| **MIG** | 873.8  (0.000-  1532) | 550.3  (0.000-  572.4) | 0.0002 | 0.68  (0.59-0.78) | < 587.8 | 81  (71-88) | 59  (46-71) |
| **TNF RII** | 3304  (2476-  4609) | 2220  (1759-  3542) | <0.0001 | **0.70**  **(0.61-0.79)** | < 2378 | 59  (48-69) | 80  (68-89) |
| **IP-10** | 105.2  (60.48-  203.1) | 50.95  (30.07-  84.08) | <0.0001 | **0.73**  **(0.64-0.82)** | < 70.56 | 71  (60-79) | 68  (55-79) |
| **I-309** | 7.415  (4.248-  12.04) | 0.000  (0.000-  4.330) | <0.0001 | **0.80**  **(0.73-0.88)** | < 3.925 | 74  (64-83) | 79  (66-87) |

**Table 6: Least Squared Mean values of biomarkers detected in plasma samples of TB patients at baseline. week 2. Month 2 and Month 6.** Data obtained from TB patients enrolled in Norway only are shown. Biomarkers with an overall significant difference and those which showed a trend in change at the end of treatment are represented. The mean values shown and 95% confidence interval (in brackets) are the least squared means. Mixed model repeated measures analysis of variance with the Fischer’s Least Significant Difference post hoc testing was used for analysis. Significant p values between timepoints are highlighted in bold. Bsl: Baseline. Wk2: Week 2. M2: Month 2. M6: Month 6

|  | **Timepoint** | | | | **P value** | | | |
| --- | --- | --- | --- | --- | --- | --- | --- | --- |
| **Marker** | **Baseline** | **Week 2** | **Month 2** | **Month 6** | **Bsl vs Wk2** | **Bsl vs M2** | **Bsl vs M6** | **Bsl vs Wk2**  **vs M2 vs M6** |
| ADAMTS13 | 101379.3  (95304.4-  107454.3) | 99067  (92214.8-  105919.1) | 111981.4  (105289.8-  118673.1) | 116552.1  (109418.4-  123685.8) | 0.46 | **<0.01** | **<0.01** | **<0.01** |
| ApoA-1 | 227.7  (208-247.2) | 241.4  (220.2-262.5) | 244.9  (224.1-265.7) | 261  (239.2-282.9) | 0.09 | **0.03** | **<0.01** | **<0.01** |
| CCL18 | 334.3  (315.2-353.3) | 338.1  (317.1-359.1) | 328.7  (308.1-349.3) | 300.7  (278.8-322.5) | 0.67 | 0.52 | **<0.01** | **<0.01** |
| CFB | 157.2  (148.8-165.7) | 156.9  (147.8-165.9) | 153.7  (144.8-162.7) | 148.1  (138.8-157.5) | 0.92 | 0.29 | **0.01** | 0.06 |
| CRP | 29.5  (27.9-31.1) | 28.4  (26.6-30.1) | 27.2  (25.5-28.9) | 25.1  (23.3-26.9) | 0.07 | **<0.01** | **<0.01** | **<0.01** |
| Ferritin | 8.8  (8.6-8.9) | 8.7  (8.5-8.8) | 8.5  (8.3-8.7) | 8.2  (8.0-8.4) | 0.13 | **<0.01** | **<0.01** | **<0.01** |
| G-CSF | 143.2  (134.5-152) | 134.2  (124.0-144.4) | 133.8  (123.9-143.6) | 127.2  (116.3-138) | 0.10 | 0.07 | **0.01** | **0.04** |
| I-309 | 15.2  (12.5-17.9) | 15.1  (12.1-18.1) | 10.2  (7.3-13.2) | 5.1  (1.9-8.2) | 0.91 | **<0.01** | **<0.01** | **<0.01** |
| ICAM-1 | 1244.6  (1175.2-1313.9) | 1225.8  (1152.2-1299.5) | 1222.8  (1150.0-  1295.5) | 1172  (1096.4-1247.5) | 0.45 | 0.37 | **0.01** | 0.05 |
| IFN-g | 19.1  (14-24.2) | 12.8  (7.0-18.6) | 10.2  (4.5-15.8) | 9.5  (3.4-15.5) | **0.02** | **<0.01** | **<0.01** | **<0.01** |
| IL-12p70 | 34.8  (22.3-47.2) | 34.7  (22.1-47.2) | 32.4  (19.9-45) | 31  (18.4-43.6) | 0.93 | 0.06 | **0.01** | **0.02** |
| IL-1Ra | 10.8  (10.3-11.3) | 10.7  (10.1-11.2) | 9.8  (9.3-10.4) | 9.5  (8.9-10.1) | 0.63 | **<0.01** | **<0.01** | **<0.01** |
| IL-2 | 42.4  (28.1-56.7) | 39.6  (23.1-56.1) | 29.4  (13.3-45.4) | 14.3  (3.2-31.8) | 0.74 | 0.12 | **<0.01** | **0.01** |
| IL-2Ra | 12.6  (12.2-13.1) | 12.5  (12.0-13) | 12  (11.5-12.5) | 11  (10.5-11.5) | 0.45 | **<0.01** | **<0.01** | **<0.01** |
| IL-4Ra | 43.5  (38.1-48.8) | 45.5  (39.6-51.4) | 37.7  (32-43.5) | 36.2  (30.1-42.3) | 0.42 | **0.02** | **0.01** | **<0.01** |
| IL-8 | 2  (1.1-2.8) | 1.8  (0.8-2.7) | 1.6  (0.7-2.6) | 0.6  (0.4-1.7) | 0.71 | 0.49 | **0.02** | 0.11 |
| IP-10 | 169.3  (143.8-194.8) | 158.9  (130-187.7) | 142.2  (114-170.3) | 111.2  (80.9-141.5) | 0.45 | **0.04** | **<0.01** | **<0.01** |
| MCP-1 | 208.3  (186.7-229.8) | 228.4  (204-252.8) | 241.3  (217.5-265.1) | 253.9  (228.3-279.5) | 0.08 | **<0.01** | **<0.01** | **<0.01** |
| MDC | 895.4  (813.3-977.6) | 866.3  (775-957.7) | 789.8  (700.3-879.2) | 771.6  (676.3-866.9) | 0.47 | **0.01** | **<0.01** | **<0.01** |
| MIG | 11.4  (9.7-13) | 11.4  (9.6-13.3) | 9.8  (8-11.6) | 8.2  (6.3-10.2) | 0.93 | 0.08 | **<0.01** | **<0.01** |
| MIP-1b | 116.4  (92.2-140.7) | 117.9  (92-143.8) | 117.5  (92-143) | 95.2  (68.6-121.7) | 0.87 | 0.91 | **0.03** | 0.07 |
| MMP-1 | 16.5  (15.6-17.4) | 16.2  (15.1-17.2) | 15.4  (14.4-16.4) | 14  (12.9-15.1) | 0.45 | **0.02** | **<0.01** | **<0.01** |
| MMP-2 | 8386.3  (7722.6-9050) | 8989.1  (8278.9-  9699.2) | 9472.2  (8771.7-  10172.8) | 10215.9  (9485.4-  10946.4) | **0.02** | **<0.01** | **<0.01** | **<0.01** |
| NCAM-1 | 5559.4  (5148.5-5970.3) | 5585.2  (5157.6-  6012.8) | 6064.6  (5640.4  6488.8) | 6345.9  (5910.9-  6780.9) | 0.83 | **<0.01** | **<0.01** | **<0.01** |
| Pentraxin3 | 16.3  (15.2-17.5) | 15.7  (14.3-17) | 14.6  (13.3-15.9) | 13.1  (11.7-14.5) | 0.36 | **0.01** | **<0.01** | **<0.01** |
| Procalcitonin | 51.1  (45.2-56.9) | 48.5  (42-55) | 43  (36.6-49.4) | 41  (34.3-47.8) | 0.37 | **<0.01** | **<0.01** | **<0.01** |
| SAA | 19.4  (18.3-20.5) | 18  (16.7-19.2) | 16.4  (15.2-17.7) | 15.1  (13.8-16.5) | **0.03** | **<0.01** | **<0.01** | **<0.01** |
| sCD40L | 29  (27.4-30.6) | 29.3  (27.4-31.1) | 28.8  (27-30.6) | 26.9  (25-28.9) | 0.79 | 0.83 | **0.03** | 0.10 |
| TNF-a | 6.4  (4.4-8.4) | 5.8  (3.6-8) | 3.6  (1.4-5.7) | 2.1  (0.2-4.3) | 0.52 | **<0.01** | **<0.01** | **<0.01** |
| TNFR II | 38.2  (35.9-40.5) | 39  (36.5-41.5) | 37.3  (34.9-39.8) | 34.5  (31.9-37) | 0.37 | 0.31 | **<0.01** | **<0.01** |
| VCAM-1 | 374.8  (359.5-390.2) | 372.5  (355-389.9) | 367.3  (350.3-384.4) | 356  (337.6-374.3) | 0.78 | 0.36 | **0.04** | 0.19 |

# Supplementary Figures


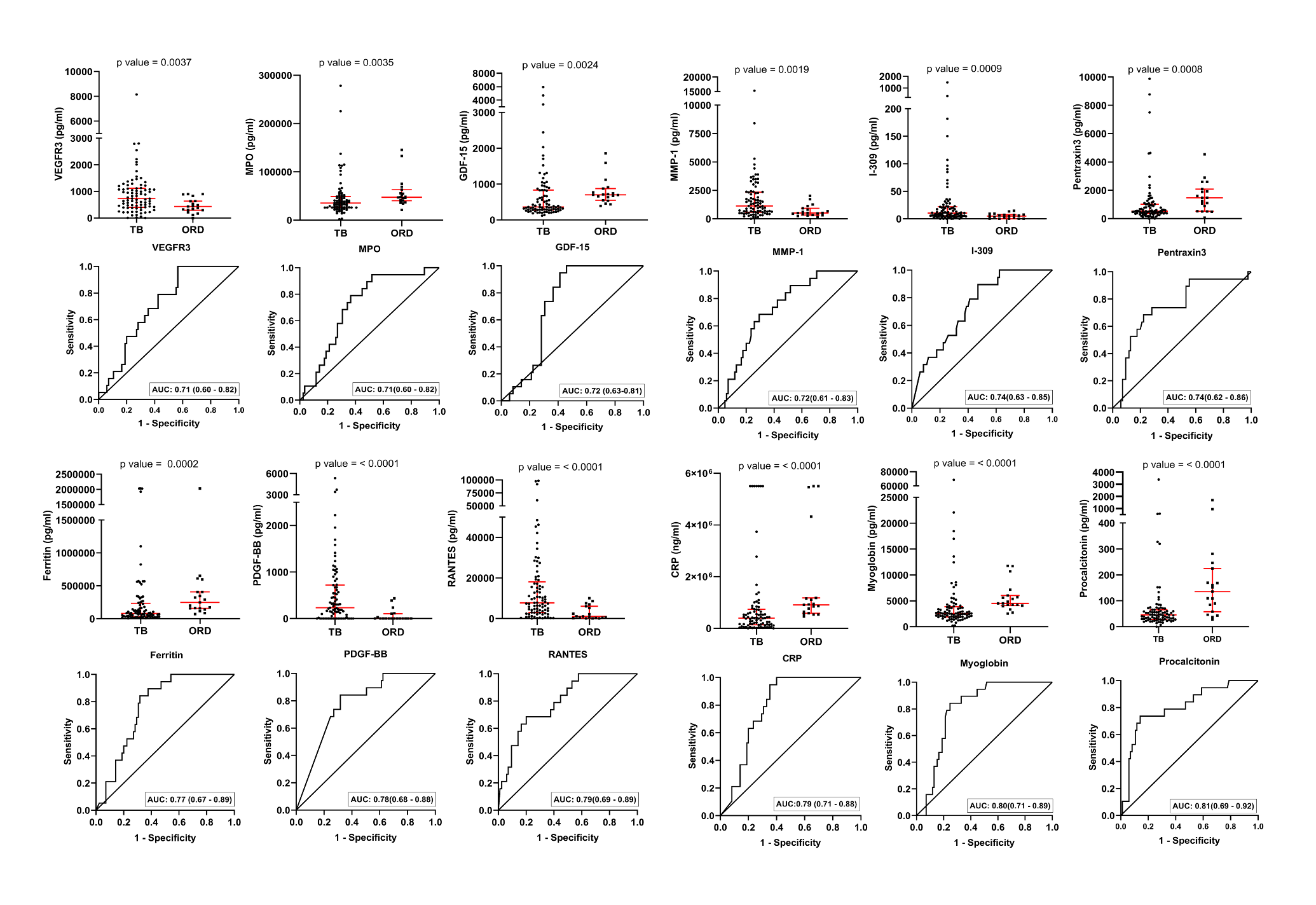


**Figure 1:** Scatter plots and ROC curves showing the concentrations of biomarkers and their accuracies in diagnosing TB disease in the Norwegian cohort. Representative plots for markers that discriminated between groups with AUC ≥ 0.70 are shown. The error bars in the scatter plots represent the median with interquartile range.


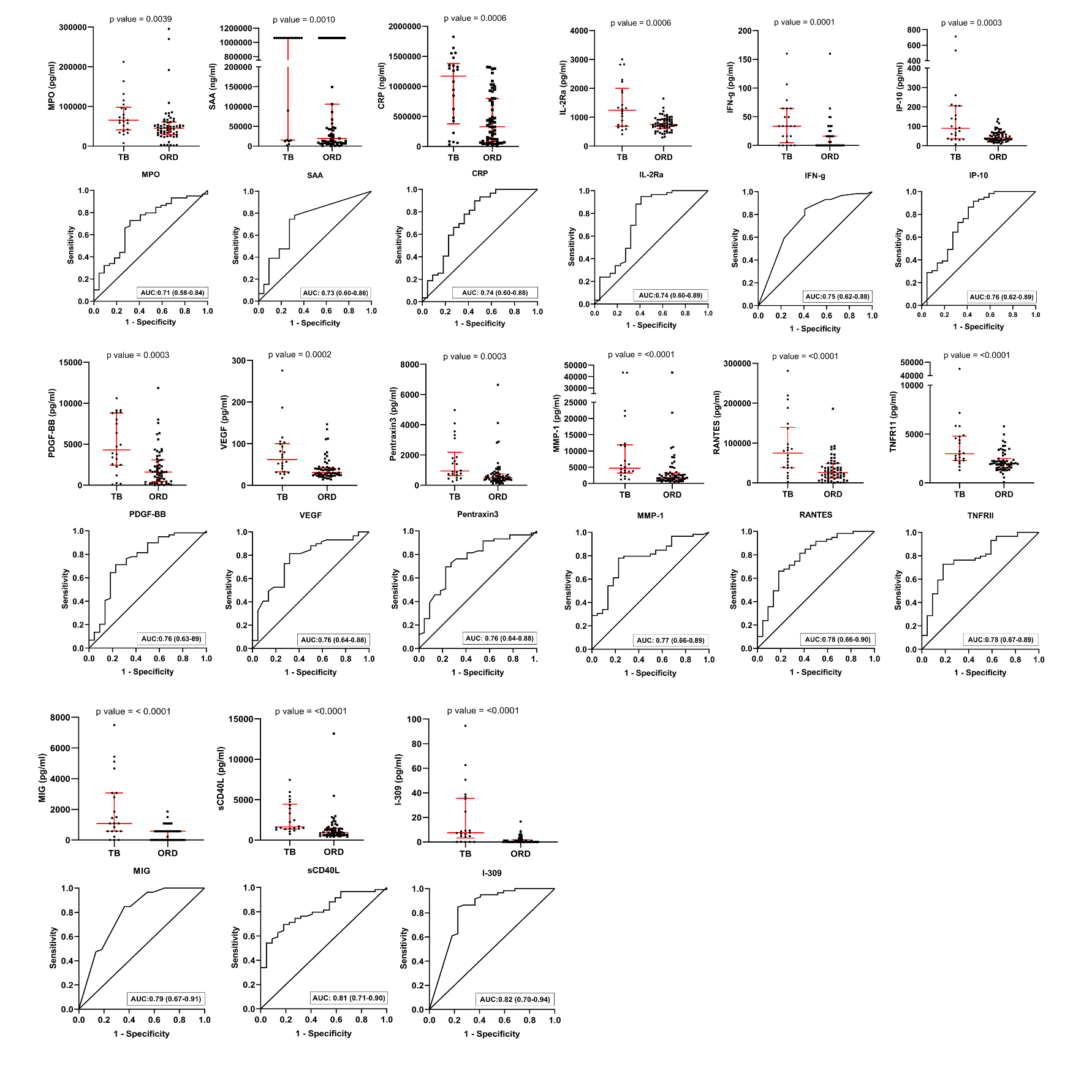


**Figure 2:** Scatter plots and ROC curves showing the concentrations of biomarkers and accuracies in diagnosing TB disease in South African study participants. Representative plots are shown for markers that discriminated between groups with AUC ≥ 0.70. The error bars in the scatter plot plots represent the median with interquartile range.


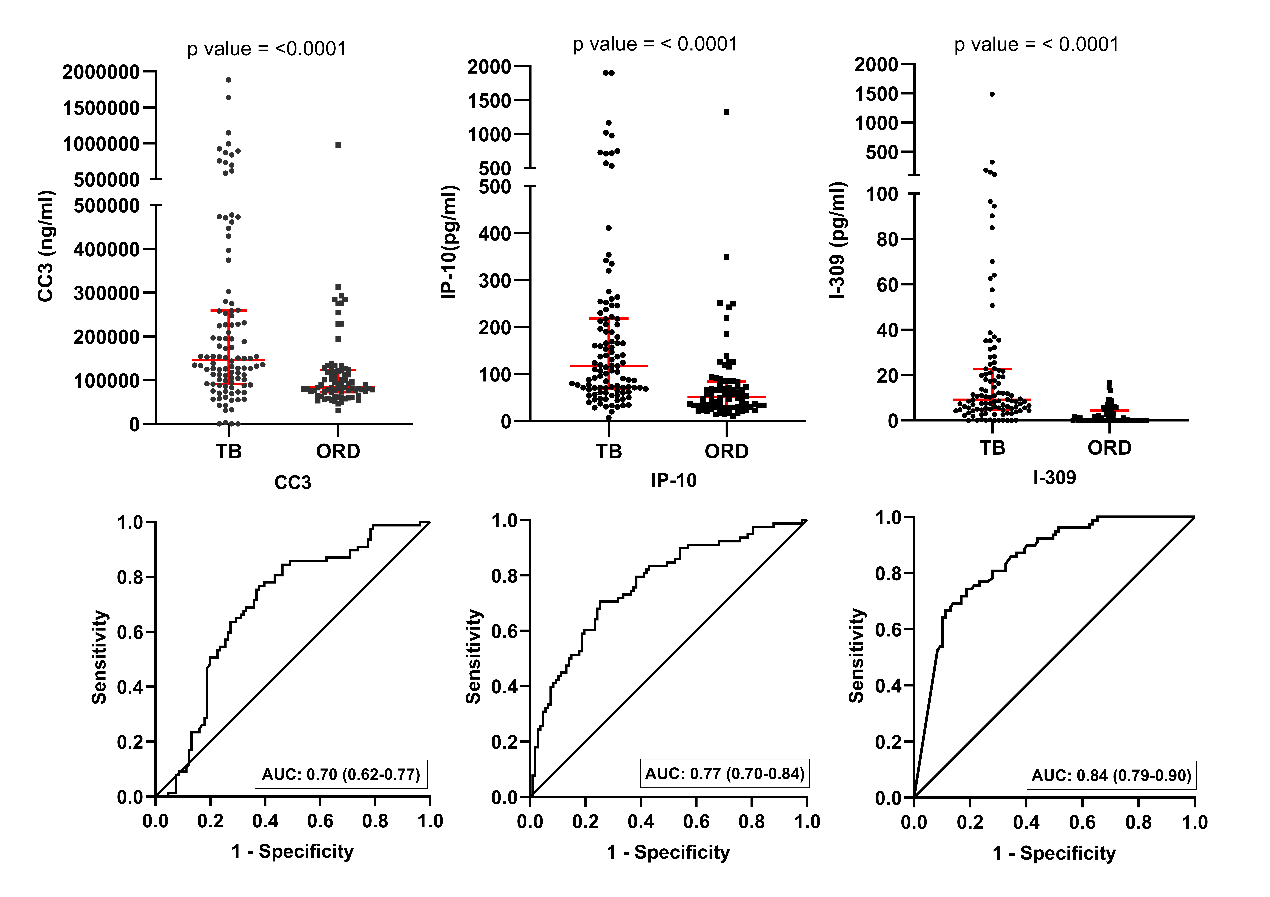


**Figure 3:** Scatter plots and ROC curves showing the concentrations of biomarkers in diagnosing TB disease in study participants. irrespective of geographical setting. Representative plots are shown for markers that discriminated between groups with AUC ≥ 0.70. when Norwegian (including EPTB patients) and South African study participants were combined. The error bars in the scatter plots represent the median with interquartile ranges.
